# Age Worsens the Cognitive Phenotype in Mice Carrying the Thr92Ala-DIO2 Polymorphism

**DOI:** 10.3390/metabo12070629

**Published:** 2022-07-08

**Authors:** Fernanda B. Lorena, Juliana M. Sato, Beatriz Martin Coviello, Alexandre J. T. Arnold, Alice Batistuzzo, Laís M. Yamanouchi, Eduardo Dias Junior, Bruna P. P. do Nascimento, Tatiana de L. Fonseca, Antonio C. Bianco, Miriam O. Ribeiro

**Affiliations:** 1 Developmental Disorders Program, Center for Biological Sciences and Health, Mackenzie Presbyterian University, Sao Paulo 01302-907, SP, Brazil; fee.lorena@gmail.com (F.B.L.); ju-sato@hotmail.com (J.M.S.); beatrizm.coviello@gmail.com (B.M.C.); alearnold@gmail.com (A.J.T.A.); abatistuzzo@uchicago.edu (A.B.); laism.yamanouchi@gmail.com (L.M.Y.); eduardo.dias@mackenzie.br (E.D.J.); bruna.pascarelli@gmail.com (B.P.P.d.N.); 2 Postgraduate Program in Translational Medicine, Department of Medicine, Paulista School of Medicine, Federal University of Sao Paulo, Sao Paulo 04021-001, SP, Brazil; 3 Section of Adult and Pediatric Endocrinology, Diabetes and Metabolism, University of Chicago, Chicago, IL 60637, USA; tatianafonseca@uchicago.edu (T.d.L.F.); abianco1@uchicago.edu (A.C.B.)

**Keywords:** thyroid hormone, cognition, type 2 deiodinase

## Abstract

The Thr92Ala-Dio2 polymorphism has been associated with reduced cognition in 2-month-old male mice and increased risk for cognitive impairment and Alzheimer’s disease in African Americans. This has been attributed to reduced thyroid hormone (TH) signaling and endoplasmic reticulum (ER) stress in the brain. Here we studied the Thr92Ala-Dio2 mouse model and saw that older male mice (7–8-month-old) exhibited a more severe cognition impairment, which extended to different aspects of declarative and working memories. A similar phenotype was observed in 4–5-month-old female mice. There were no structural alterations in the prefrontal cortex (PFC) and hippocampus of the Thr92Ala-Dio2 mouse. Nonetheless, in both male and female PFC, there was an enrichment in genes associated with TH-dependent processes, ER stress, and Golgi apparatus, while in the hippocampus there was additional enrichment in genes associated with inflammation and apoptosis. Reduced TH signaling remains a key mechanism of disease given that short-term treatment with L-T3 rescued the cognitive phenotype observed in males and females. We conclude that in mice, age is an additional risk factor for cognitive impairment associated with the Thr92Ala-Dio2 polymorphism. In addition to reduced TH signaling, ER-stress, and involvement of the Golgi apparatus, hippocampal inflammation and apoptosis were identified as potentially important mechanisms of a disease.

## 1. Introduction

Hypothyroidism is a common disease that affects between 15 and 20 million individuals in the US and hundreds of millions worldwide [1]. Autoimmune destruction or surgical removal of the thyroid gland results in lower circulating levels of thyroid hormones (TH), which leads to the systemic symptoms exhibited by patients with hypothyroidism—particularly cognitive and metabolic symptoms. The standard of care for the treatment of hypothyroidism is to restore TH levels to normal with daily tablets of levothyroxine (LT4) [2]. The rationale for treatment with LT4 is that, after absorption, T4 is activated to T3 via deiodinases expressed in multiple tissues—T3 being the active TH. While this rationale is commonsensical, it places the deiodination process at the center of the treatment with LT4. In other words, any deiodinase impairments can potentially reduce the effectiveness of treatment with LT4 [3].

Types 1 and 2 deiodinases (D1 and D2) can convert T4 to T3. D1 is expressed predominantly in the liver and kidneys. In contrast, D2 expression is predominantly in the brain, pituitary gland, and brown adipose tissue, but can also be found in large tissues such as skeletal muscle and skin, and also in the endothelial cells. The D2 pathway is considered the most relevant in humans given that the inhibition of the D1 enzyme with propylthiouracil in LT4-treated patients only reduced T3 serum levels by approximately 20% [4].

In recent years, a common polymorphism in the gene encoding D2 (DIO2) has attracted attention because the substitution of Thr for Ala in position 92 reduces the D2 catalytic activity by approximately 20% [5,6]. Hence the concern that carriers of this polymorphism could be at risk of reduced effectiveness of treatment with LT4 is justified. Although mice carrying the Ala92-Dio2 polymorphism are systemically euthyroid, they do exhibit changes in gene expression in different areas of the brain that are compatible with the localized reduction in TH action [6].

In addition, there is evidence, both in mice [6] and humans [7,8], that carriers of the DIO2 polymorphism exhibit stress of the endoplasmic reticulum (ER) and a transcriptome compatible with the degenerative disease of the central nervous system (CNS). The ER stress results from the accumulation of the Ala-D2 enzyme, which escapes the normal turnover pathway through the ubiquitin-proteasome system. Ala-D2 accumulates ectopically in the Golgi apparatus, instead of being retained in the ER like the Thr92-D2 enzyme. This seems to be clinically relevant given that African (but not European) Americans that carry the Ala92-DIO2 polymorphism are at greater risk of developing cognitive impairment and Alzheimer’s disease [7,8].

Male mice carrying the Ala92-Dio2 polymorphism exhibit an important cognitive phenotype—impaired short-term memory—associated with reduced mobility and increased sleeping time [6]. Given that the prevalence of hypothyroidism is higher in women and it increases with age, here we investigated the impact of the Ala92-Dio2 polymorphism in older mice. We also investigated whether female mice carrying the Ala92-Dio2 polymorphism exhibited a cognitive phenotype.

## 2. Results

### 2.1. Older Age Intensifies the Cognitive Phenotype in Male Ala92-Dio2 Mice

**Figure 1:**
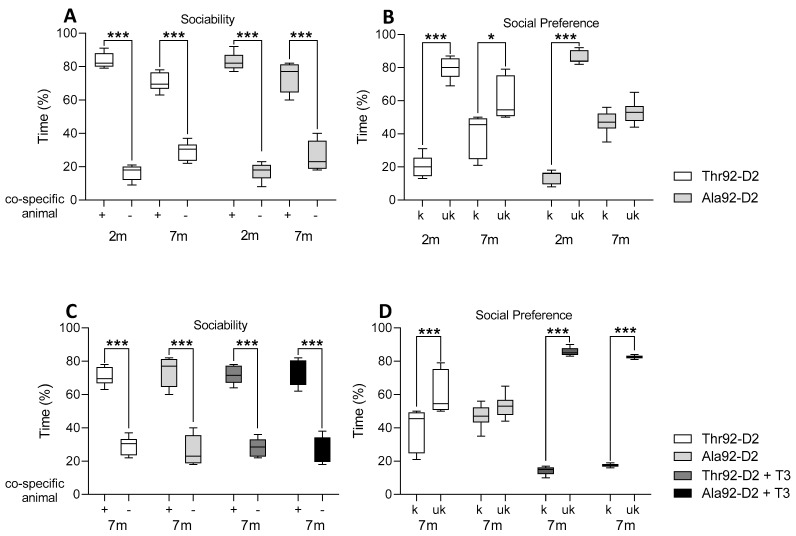
Cognitive tests in 2–3 (2 m) and 7–8-month-old (7 m) male mice. The social recognition test are displayed as time (% of total time) to assess sociability (**A**,**C**) and social preference (**B**,**D**) for Thr92-Dio2 and Ala92-Dio2 mice and mice treated with T3 for ten days; in each plot, “+” represents a co-specific animal and “−“ represents an empty cage and k represents a familiar mouse and uk represents an unknown mouse; n = 6–7/group. * *p* < 0.05 and *** *p* < 0.001 as calculated through the two-way ANOVA followed by the Bonferroni post-test. These experiments were repeated once with similar results; only one set of results is shown in the figure.

After the mice were transferred and a new colony was established in our laboratory in Sao Paulo, Brazil, we wanted to confirm that their phenotype remained. Our previous studies have identified decreased 24 h-mobility in the young (2-month-old) Ala92-Dio2 mouse [6]. Here we also detected a ~25% reduction in mobility in the 2–3-month-old male Ala92-Dio2 mice during the OF test, but the difference did not reach statistical significance (Table 1). In addition, knowing that the Ala92-Dio2 male mouse exhibits impaired short-term memory [6], here we also expanded the investigation to other memory domains of male mice but found that sociability (Figure 1A) and social preference (social recognition test) (Figure 1B), working and visual-spatial memories (hole board test) were preserved (Table 1).

To determine whether the Ala92-Dio2 phenotype could be worsened by age, we evaluated another set of male mice at ages 7–9-months. There was decreased mobility in Ala92-Dio2 mice when compared to Thr92-Dio2 mice (Table 1), but the speed at which these mice moved around the cage was not different (Table 1). The visuospatial memory was not affected in the older mice, with no differences between genotypes (Table 1). While the working memory was also not affected by age in Thr92-Dio2 mice, the older Ala92-Dio2 mice did exhibit an impairment in this parameter (Table 1). Sociability was preserved in the older Ala92-D2 mice (Figure 1A) but they exhibited a blunted ability to recognize new mice (Figure 1B).

Given that in previous studies the phenotype observed in the young male Ala-Dio2 mouse was rescued by treatment with LT3 [6], we also tested whether this would happen with older male Ala92-Dio2 mice. Indeed, we noticed that treatment with LT3 for 10 days (1 µg LT3/mouse) did not affect visuospatial memory but improved working memory in Ala92-Dio2 mice, eliminating the differences between genotypes (Table 2). At the same time, treatment with LT3 did not affect sociability (Figure 1C) but improved declarative memory in both Thr92-Dio2 and Ala92-Dio2 mice, eliminating the differences between the genotypes (Figure 1D). Treatment with LT3 reduced mobility and velocity in Thr92-Dio2 but not in Ala92-Dio2 mice, and no differences between genotypes were observed after LT3 treatment (Table 2).

Sections of the hippocampus and retrosplenial cortex were prepared and stained with cresyl violet for further analyses (Figure S1). The slides were scanned, and the digitized images were analyzed for the area and density of neurons using the QuPath software. The CA1, CA2, CA3, and dentate gyrus hippocampal areas were analyzed (Table 3, as well as the ventral and dorsal components of the retrosplenial cortex (Table 4). Only the hippocampus of the Ala92-Dio2 mice exhibited a minor decrease in the neuronal density in the CA3 area (Table 4).

### 2.2. The Phenotype Associated with Ala92-Dio2 Polymorphism Is Modified in Female Mice

**Figure 2:**
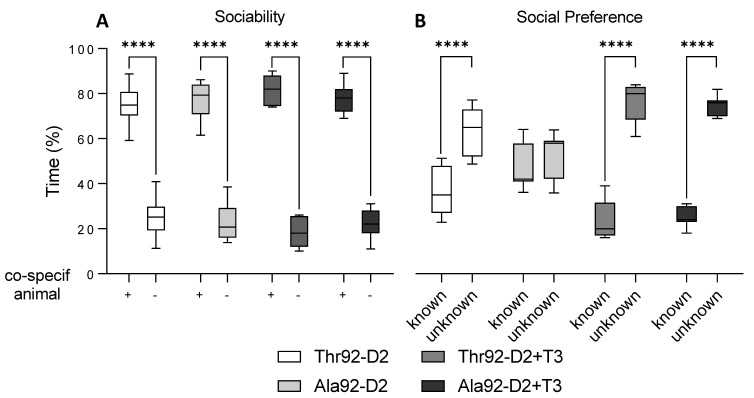
Cognitive tests in 3–4-month-old female mice. The social recognition tests are displayed as time (% of total time) to assess sociability (**A**) and social preference (**B**) for Thr92-Dio2 and Ala92-Dio2 mice and mice treated with T3 for ten days; in each plot, “+” represents a co-specific animal and “−” represents an empty cage, and k represents a familiar mouse and uk represents an unknown mouse; n = 6–7/group. **** *p* < 0.001 as calculated through the two-way ANOVA followed by the Bonferroni post-test. These experiments were repeated once with similar results; only one set of results is shown in the figure.

3–4-month-old female Ala92-Dio2 mice exhibited ambulatory activity similar to Thr92-Dio2 and preserved visual-spatial memory, but they had a substantial impairment in working memory (Table 3). Although the Ala92-Dio2 females exhibited normal sociability (Figure 2A), they were unable to differentiate the new co-specific mouse from the familiar one, indicating an impairment in the declarative memory (Figure 2B). Here we also asked whether treatment with LT3 could improve this phenotype. Treatment with LT3 for 10 days (1 µg LT3/mouse) improved visuospatial memory but worsened working memory in mice of both genotypes (Table 4). Treatment with LT3 did not affect sociability (Figure 2A) but fully restored the declarative memory in the Ala92-D2 females (Figure 2B). Of note, the differences in working memory between genotypes disappeared after LT3 treatment owing to an increase in the number of errors in both groups (Table 3). While it is not clear why that happened, other investigators observed a similar effect of LT3 on the working memory [9,10].

The analysis of sections of the hippocampus and retrosplenial cortex of the female mice (Figure S2) revealed a decrease in the area of the Ala92-Dio2 hippocampal CA1 and dentate gyrus (Table 5). In addition, there was an increase in neuronal density in the CA1 area (Table 5).

### 2.3. Transcriptome Analysis

**Figure 3:**
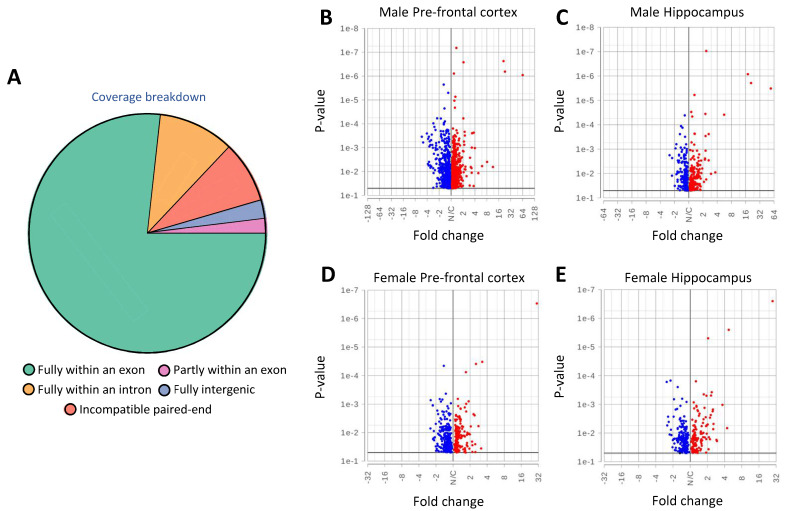
Pre-frontal cortex and hippocampus gene expression profile in Ala92-Dio2 male and female mice. (**A**) RNA-seq coverage breakdown; distribution reflects the average of all samples; (**B**) Pre-frontal cortex differential gene expression (Volcano plot) in Ala92-Dio2 male vs. Thr92-Dio2 male; each point represents the average of 4 mice for each transcript; shown in red are upregulated genes with *p* < 0.05; shown in blue are downregulated genes with *p* < 0.05; n = 4–6/group (**C**) Same as A, except that shown is male hippocampus; (**D**) Same as A, except that shown, is the female prefrontal cortex; (**E**) Same as A, except that shown is the female hippocampus. The statistical analysis was done using one-way ANOVA.

Next, we wished to characterize the molecular basis of the phenotypes associated with carrying the Thr92Ala-Dio2 polymorphism. Hence, we performed RNA-seq analysis of the PFC and hippocampus of male and female mice (Figure 3A–E).

In the older males, carrying the Ala92-Dio2 polymorphism was associated with the differential expression of 1191 genes in the PFC and 431 in the hippocampus as compared to controls (Figure 3B,C; Tables S1 and S2. The biological interpretation of these changes was studied using gene set enrichment analysis (GSEA). In the PFC, the Ala92-Dio2 males exhibited 1294 enriched gene sets (Table S3), whereas, in the hippocampus, 545 gene sets were enriched (Table S4). Our previous studies in discrete brain areas of younger Ala92-Dio2 males revealed enrichment of gene sets related to the Golgi apparatus and the ubiquitin-proteasome system [6]; here also, 7 related gene sets were identified in the PFC and 6 in the hippocampus (Tables S3 and S4). However, we also identified additional gene sets enriched in the Ala92-Dio2 PFC, 36 sets related to neuronal signaling, 23 sets related to neuroplasticity, and 13 sets related to cognition (Table S5). At the same time, the hippocampus of the same mice exhibited 4 gene sets related to neuronal signaling, 4 to neuroplasticity, 2 gene sets related to behavior, and 7 gene sets related to apoptosis or inflammation (Table S6). These new findings suggest that the alterations in the older Ala92-Dio2 mouse brain are indeed more substantial than those originally described in the younger mice.

Female mice carrying the Ala92-Dio2 polymorphism also exhibited a significant transcriptome alteration. There were 475 genes differentially expressed in the PFC and 324 in the hippocampus as compared to controls (Figure 3D,E; Tables S7 and S8). In the PFC, 517 gene sets were enriched in the Ala92-Dio2 females (Table S9), whereas in the hippocampus, 340 gene sets were enriched (Table S10). Similar to the males, here too there was an enrichment of Golgi-related or ubiquitin-proteasome system-related gene sets in the PFC (7 gene sets) and the hippocampus (10 gene sets) (Tables S9 and S10). Carrying the Ala92-Dio2 polymorphism was associated with an enrichment of 13 gene sets related to neuronal signaling in the PFC, 10 gene sets related to neuroplasticity, and 2 related to apoptosis (Table S5). At the same time, the hippocampus of the same mice exhibited 4 gene sets related to neuroplasticity, 2 to behavior, and 9 gene sets related to apoptosis or inflammation (Table S6).

**Figure 4:**
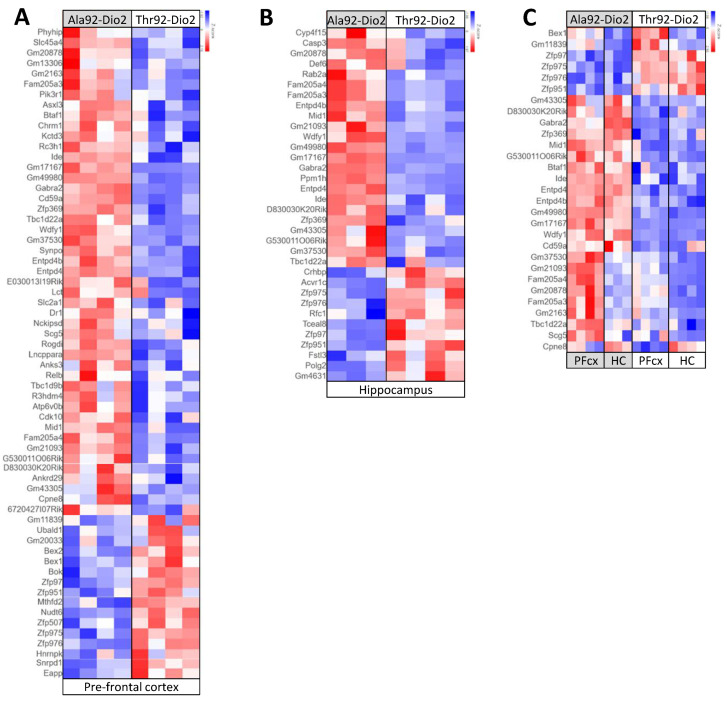
Heat map of genes from Ala92-Dio2 mice shown in Figure 3. (**A**) heat map of 63 genes (47-up and 16-down regulated genes) differentially expressed in Ala92-Dio2 pre-frontal cortex that overlapped in male and female mice; (**B**) same as A, except that shown is 34 genes (23-up and 11-down regulated genes) that overlapped in the hippocampus of male and female mice; and (**C**) Same as A, except that shown is 29 genes (23-up and 6-down regulated) overlapped in the pre-frontal cortex and hippocampus of male and female mice; relative gene expression is indicated by the degree of color saturation (red: higher level; blue: lower level).

Throughout the transcriptome analyses, we noticed some degree of overlap between PFC and hippocampus in males and females concerning genes that were enriched or impoverished in mice carrying the Ala92-Dio2 polymorphism. To explore this further, we used Venn diagrams to identify the overlapping genes, having found 63 common genes (47-up and 16-down regulated genes) in the PFC of males and females, and 34 common genes (23-up and 11-down regulated genes) in the hippocampus (Figure 4A,B). Overall, 29 genes overlapped in the PFC and hippocampus of male and female mice (23-up and 6-down regulated) (Figure 4C; Table S11).

**Figure 5:**
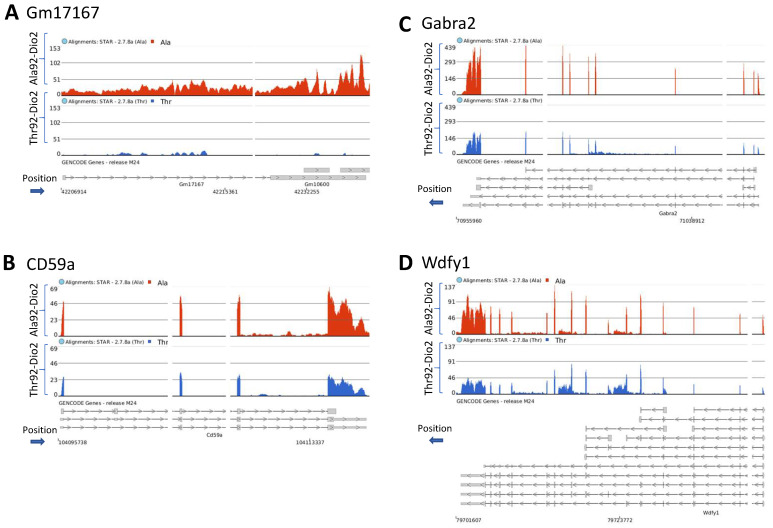
Gene expression coverage in the Ala92-Dio2 mice. (**A**–**D**) browser Integrative Genomics Viewer (IGV)-display of transcript areas of selected genes (4 representative genes out of a total of 29 genes that overlapped in the pre-frontal cortex and hippocampus of male and female mice) that are upregulated in Ala92-Dio2; the gene names are indicated at the top of each panel along with the blue arrow indicating the direction of transcription; the red tracks reflect the coverage for the Ala92-Dio2 RNA-seq data, and the blue tracks reflect the Thr92-Dio2 Rna-seq data.

Of these genes, 10 do not have a canonical name but the top gene Gm17167 (Figure 5A) is known for being reduced in the hippocampus of mice exposed to fear [11]. The remaining genes included Cd59a (Figure 5B; a crucial mediator of neuroinflammation and neurodegeneration after traumatic brain injury) [12], Gabra2 (Figure 5C; GABA type-A receptors, critical in modulating inhibitory synaptic function) [13], Wdfy1 (Figure 5D; involved in inhibition of neurogenesis and development of neurodegenerative diseases) [14], Zfp369 transcriptional repressor involved in apoptosis), Ng4 (important in the development, maintenance, and repair of both the central nervous system (CNS) [13] and peripheral nervous system (PNS) [15], all upregulated in the Ala92-Dio2 mice. In addition, BEX1 (confers resistance to Amyotrophic Lateral Sclerosis (ALS) [16], was downregulated in the Ala92-Dio2 mice.

## 3. Discussion

The present studies revealed that the phenotype of mice carrying the Thr92Ala-Dio2 polymorphism includes impairment in memory processes that involve the PFC (working memory) and the hippocampus (declarative memory). Whereas the transcriptome analyses of both regions indicated the involvement of the Golgi apparatus and ER stress, the PFC exhibited specific changes in gene expression connected with neuronal signaling, neuroplasticity, and learning and cognition. In contrast, the predominant changes in the hippocampal transcriptome were connected with inflammation and apoptosis. Here we identified age as a risk factor for a more severe cognitive phenotype in 7–8-month-old male mice carrying the Thr92Ala-Dio2 polymorphism. Female mice also exhibited a distinct cognitive phenotype at age 4–5-month-old.

The Thr92-AlaD2 enzyme is catalytically less active. Thus, it made sense that one of the mechanisms of the disease previously identified in the young males was a subtle reduction in TH signaling in the striatum, amygdala, and PFC [6]. Indeed, the changes in PFC gene expression observed in the present investigation include two major processes known to be TH-dependent, i.e., neuronal signaling and neuroplasticity [17]. That reduced TH signaling plays a role in the Thr92Ala-Dio2 phenotype is also supported by the observation that short-term administration of LT3 to both Thr92Ala-Dio2 males resolved their cognitive phenotype (in females it resolved only the declarative memory), confirming that reduced T3 signaling is an important disease mechanism in the brain of these animals.

The involvement of the ubiquitin-proteasomal system, ER stress, and inflammation seem to be key to the development of the phenotype associated with the Thr92Ala-Dio2 polymorphism given that treatment with chemical chaperones improved the phenotype [6]. Indeed, using RNA-seq of the PFC and hippocampus, here we consistently identified 6–10 gene sets related to these processes that were enriched in both Thr92Ala-Dio2 CNS areas. ER stress and inflammation were also identified in the Thr92Ala-Dio2 in the brains of European and African American carriers of the polymorphism [7,8].

Previously we had observed that the Ala-D2 protein has a prolonged half-life (less susceptible to ubiquitination and proteasomal degradation) and ectopically accumulates in the Golgi apparatus. Cells expressing the Ala-D2 protein exhibit clear signs of ER stress that can be resolved by treatment with chemical chaperones, suggesting that the Ala-D2 protein is misfolded [6]. Indeed, it is broadly recognized that protein misfolding leading to ER stress is a key factor in the pathogenesis of neurodegenerative diseases [17]. For example, in Huntington’s disease (HD), the mutant huntingtin has been implicated in CNS ER stress and consequent cellular toxicity [18]. There is also clear evidence that ER stress resulting from the accumulation of intracellular neurofibrillary tangles plays a critical role in neurodegeneration linked to Alzheimer’s disease [19,20]. This is relevant given that African Americans carriers of the Thr92Ala-DIO2 polymorphism have an increased likelihood of developing Alzheimer’s disease [8].

The relationship between ER stress and inflammation in the CNS is well-known [21]. Neuroinflammation involves astrocytes, the main cell type expressing Dio2 in the CNS, hence the probable site where ER stress is triggered. Given our RNA-seq findings, hippocampal neuroinflammation seems to be a plausible additional mechanism leading to impaired neurogenesis and cognition in the Thr92Ala-Dio2 mouse [22,23]. Neuroinflammatory conditions can also lead to impaired cell survival and apoptosis, compromising neuronal differentiation, and cell proliferation, all of which affect the hippocampal neurogenic process [24,25].

ER stress and neuroinflammation as a mechanism of disease in the Thr92Ala-DIO2 mouse is also supported by the worsening phenotype exhibited in the older males. The characteristic signatures of “brain aging” include, among other elements, a cumulative buildup of neuroinflammation [25], particularly in the hippocampus, cerebral cortex, and cerebellum [26,27,28]. The cumulative buildup of dysfunctional proteins and an unfolded protein response, markers of ER stress also correlate with changes in cognition [21,26].

Of note, 4–5-month-old female mice exhibited clear signs of impaired working memory and social discrimination despite a protective effect of estrogens on neuroinflammation and ER stress [29,30]. Future studies should address and clarify whether females have increased susceptibility to the Thr92Ala-Dio2 polymorphism.

## 4. Materials and Methods

### 4.1. Animals

All experiments followed the American Thyroid Association Guide to investigating TH action in rodent models [30] and were approved by the Ethics Committee at Universidade Presbiteriana Mackenzie (CEUA/UPM Nº 180-10-2019). Thr92-Dio2 and Ala92- Dio2 mice were created as described previously [6]. Briefly, mice were produced using CRISPR/Cas9 technology using C57BL/6 mice (Applied Stemcell Inc., Milpitas, CA, USA). Control mice were “humanized” replacing the native proline for threonine in position 92. This is because, in mice, position 92 in the Dio2 is a proline, while in humans is a threonine.

Experiments were performed in females and males, weighing between 25 and 30 g, kept in a 12-h light/dark cycle at 25 °C, and in collective cages with 5 mice per cage, with food and water ad libitum. One set of male mice was studied at age 2–4 months and another set at age 7–8 months to avoid the “carry over effect”, i.e., the experience of the first test impacting the way mice react during the second time around. Females were studied at ages 4–5 months. To synchronize the estrous cycle and reduce the behavioral fluctuations caused by estrogens, two days before the start of the behavioral tests, the females underwent the “Whitten effect”, which consists of exposing the females to cages containing male urine [31,32,33]. Estrous was confirmed through vaginal cytology.

### 4.2. Behavior and Cognitive Assessment

Both females and males underwent a sequence of behavioral tests in the following order: open field (OF), social recognition test (SR), and hole board test (HB).

An OF test was used to evaluate locomotor capacity. Animals were exposed to the apparatus for 10 min. All sessions were video recorded for later analysis using the EthoVision software (Noldu Inf. Tech.; Wageningen, The Netherlands). Mouse movements were analyzed for the average speed (cm/s) and distance traveled (cm) in all zones [34].

The SR test assesses sociability and declarative memory through olfactory stimuli, based on the investigative and spontaneous behavior of the mice and on the fact that an unfamiliar mouse will be more explored than a familiar mouse [35]. The SR tests were carried out in an acrylic apparatus (20 × 40 × 22 cm) and the following parameters were evaluated: (i) % of time spent with a co-specific mouse and (ii) % of the time in contact with a known and an unknown mouse.

The HB test assesses working memory and visuospatial memory and was performed in the OF test apparatus containing a platform with 9 equidistant holes, measuring 4.5 cm in diameter and 3.0 cm in depth [36,37]. Food was withdrawn the night before the first day of the test, which was carried out during the morning period (09:00–12:00 h). During the test, the mouse’s ability to remember which of the 9 equidistant holes had a bait was evaluated. During 4 consecutive days, mice were trained in 4 daily sessions (trials) of 180 s with an interval of 180 s between each session (trial) per animal. On the first day of training, the mice were habituated on the hole board platform for 10 min. For all training sessions, the same holes were baited, with a single food granule (chocolate candy). After the fourth training day, the mice had free access to food only until 6 PM, and food was subsequently withdrawn in preparation for the next day’s test. The percentage of time the mouse visits the hole without reward was considered a visuospatial memory error and the percentage of time the mouse revisited a bounty hole that he has already eaten was considered a working memory error.

As indicated, some mice received two daily T3 daily injections (1 μg/day; i.p.), the first in the morning and the second in the afternoon, for 10 consecutive days. On the fourth day of treatment, the SR test was performed and, on the seventh day, the HB test was performed. At the end of treatment, the mice were euthanized and their brains were processed for subsequent analyses [6].

### 4.3. RNA Sequencing and Analyses

Animals were euthanized by asphyxiation in a CO_2_ chamber. Immediately after killing, the brain was dissected using a magnifier lens, and the hippocampus and PFC were identified and surgically dissected. Brain areas were then snap-frozen in liquid nitrogen and stored at −80 °C for RNA analysis. RNA was isolated from 4 Thr92-Dio2 and 4 Ala92-Dio2 mouse brains for gender using the RNeasy Kit (Qiagen). RNA degradation was monitored using a BioAnalyzer (Agilent). Samples of total RNA with RIN > 9.2 were sent to the Genomic facility at the University of Chicago for library preparation and sequence. Libraries were pair-end sequenced with NovaSeqS4 (Illumina). Base calls and demultiplexing were performed with Illumina’s bcl2fastq software and a custom python demultiplexing program with a maximum of one mismatch in the indexing read. The FASTQ files were aligned to gencode vM24 transcriptome with STAR (v.2.7.8a) using the Partek flow platform (Partek Inc., St. Louis, MO, USA). All pre- and post-alignment QA/QC was performed in Partek Flow (Partek Inc.) (Figure S1). Aligned reads were quantified to the annotation model (Partek E/M) and normalized (absolute value). Following the differential analysis (ANOVA), the biological significance of the changes was interpreted using gene set enrichment analysis (GSEA; Tables S5–S8).

### 4.4. Statistical Analyses

Experimental data were submitted to statistical analysis to assess their relevance using GraphPad software. The statistical significance of the difference between the mean values was analyzed by T Student test for two groups or two-way ANOVA (OF, SR, and HB), followed by Bonferroni’s post-test for more than two variables, with a significance level of *p* < 0.05.

## 5. Conclusions

Older age and female sex are risk factors for the cognitive impairment associated with the Thr92Ala-Dio2 polymorphism in mice. Carrying the Thr92Ala-Dio2 polymorphism is associated with changes in gene expression in the PFC and hippocampus that involve the stress of the ER and Golgi apparatus, a critical disease mechanism observed in other types of degenerative diseases of the CNS. The structural studies of both areas revealed only minimal alterations, which is compatible with our findings that short-term treatment with T3 reversed most of the cognitive phenotype in both males and females. In addition, the present studies identified hippocampal inflammation and apoptosis as new disease mechanisms, both processes are also involved in other types of CNS degeneration.

## Figures and Tables

**Table 1:** Cognitive and behavioral phenotype of 2–3-month-old and 7-old-month male Ala92-Dio2 mice.

Test	Parameter	Thr92-Dio2 2–3 m	Ala92-Dio2 2–3 m	Thr92-Dio2 7 m	Ala92-Dio2 7 m
Open field	Distance (cm)	5535 ± 288.8	4399 ± 262.7	4742 ± 522.7	3251 ± 265.5 ^a^
	Velocity (cm/s)	9.8 ± 0.8	8.0 ± 0.5	10.21 ± 1.74	6.98 ± 1.08
Hole board	Visuospatial memory (% error)	32.3 ± 12.4	54.6 ± 9.1	27.4 ± 5.9	13.58 ± 7.3
	Working memory (% error)	1.63 ± 1.14	6.47 ± 1.69	3.0 ± 1.93	21.5.3 ± 3.17 ^bc^

For Open field test results of mice 2–3-month-old: Thr92 2 m-Dio2 n = 9; Ala92-Dio2 2 m n = 9 and of mice 7-old-month: Thr92-Dio2 7 m n = 6; Ala92-Dio2 7 m n = 7. For Hole board test results: Thr92-Dio2 2 m n = 12; Ala92-Dio2 2 m n = 12; Thr92-Dio2 7 m n = 12; Ala92-Dio2 7 m n = 12. “% error” is the percentage of errors made by mice while recalling the holes that contain food. Values are expressed as mean ± SEM; Statistical analysis using 2-way ANOVA followed by Bonferroni’s test. ^a^ vs. Thr92-Dio2 7 m with *p* < 0.05; ^b^ vs. Thr92-Dio2 7 m with *p* < 0.001 and ^c^ vs. Ala92-Dio2 2 m with *p* < 0.001.

**Table 2:** Cognitive and behavioral phenotype of 7-old-month male Ala92-Dio2 mice treated with T3.

Test	Parameter	Thr92-Dio2 7 m	Ala92-Dio2 7 m	Thr92-Dio2 + T3 7 m	Ala92-Dio2 + T3 7 m
Open field	Distance (cm)	4742 ± 522.7	3251 ± 265.5	2705 ± 141.6 ^a^	2096 ± 207.9
	Velocity (cm/s)	10.21 ± 1.74	6.98 ± 1.08	4.45 ± 0.23 ^b^	3.52 ± 0.35
Hole board	Visuospatial memory (% error)	27.4 ± 5.9	13.58 ± 7.3	25.8 ± 3.77	17.7 ± 4.87
	Working memory (% error)	3.0 ± 1.93	21.5.3 ± 3.17 ^c^	0.00 ± 0.00	0.00 ± 0.00 ^d^

For Open field test results of mice 7-month-old: Thr92-Dio2 n = 6; Ala92-Dio2 n = 7. For Hole board test results: Thr92-Dio2 n = 12; Ala92-Dio2 n = 12; Thr92-Dio2 + T3 n = 6; Ala92-Dio2 + T3 n = 7. “% error” is the percentage of errors made by mice while recalling the holes that contain food. Values are expressed as mean ± SEM; Statistical analysis using 2-way ANOVA followed by Bonferroni’s test. ^a^ vs. Thr92-Dio2 with *p* = 0.006; ^b^ vs. Thr92-Dio2 with *p* < 0.005; ^c^ vs. Thr92-Dio2 with *p* < 0.0001 and ^d^ vs. Ala92-Dio2 with *p*< 0.0007.

**Table 3:** Area and neuronal density of Cornus ammonis (CA1, CA2, CA3) and Dentate gyrus (DG) hippocampal areas and retro splenial cortex (RSC) of 7-month-old male Ala92-Dio2 and Thr92-Dio mice.

Male					
	Area	Parameter	Thr92-Dio2	Ala92-Dio2	*p*-Value
	**CA1**	Area (mm^2^)	117.7 ± 7.07	110.1 ± 6.54	0.464
		Neuronal density (n/mm^2^)	10.1 ± 0.24	10.1 ± 0.63	0.992
	**CA2**	Area (mm^2^)	16.6 ± 0.70	14.8 ± 1.69	0.35
		Neuronal density (n/mm^2^)	10.6 ± 0.51	9.7 ± 0.81	0.412
**Hippocampus**					
	**CA3**	Area (mm^2^)	100.3 ± 5.93	132.2 ± 16.8	0.124
		Neuronal density (n/mm^2^)	10.2 ± 0.30	8.5 ± 0.13	0.002
	**DG**	Area (mm^2^)	144.6 ± 16.39	116.9 ± 11.55	0.216
		Neuronal density (n/mm^2^)	9.8 ± 0.85	11.5 ± 0.27	0.11
	**Ventral**	Neuronal density (n/mm^2^)	7.0 ± 0.75	7.0 ± 0.23	0.957
**RSC**					
	**Dorsal**	Neuronal density (n/mm^2^)	7.4 ± 0.82	6.8 ± 0.77	0.769

Values are expressed as mean ± SEM. Statistical analysis using Student’s *t*-test. (Thr92-Dio2 n = 6; Ala92-Dio2 n = 4). Neuronal density is shown as neurons/mm^2^; these experiments were repeated once with similar results; only one set of results in shown in the table.

**Table 4:** Cognitive and behavioral phenotype of 3–4-old-month female Ala92-Dio2 mice treated with T3.

Test	Parameter	Thr92-Dio2	Ala92-Dio2	Thr92-Dio2 + T3	Ala92-Dio2 + T3
Open field	Distance (cm)	1909 ± 305.3	1679 ± 200.9	1065 ± 37.7	942. ± 127.8
	Velocity (cm/s)	4.74 ± 0.49	4.56 ± 0.51	5.52 ± 0.86	4.66 ± 0.56
Hole board	Visuospatial memory (% error)	57.4 ± 3.5	50.3 ± 3.11	12.8 ± 7.14 ^a^	15.57 ± 6.16 ^b^
	Working memory (% error)	0 ± 0	10.3 ± 1.7 ^c^	19.8 ± 5.49 ^d^	21.86 ± 5.27 ^e^

For Open field test results of female mice 3–4-month-old: Thr92-Dio2 n = 10; Ala92-Dio2 n = 10 and for T3 treated female: Thr92-Dio2 + T3 n = 6; Ala92-Dio2 + T n = 7. For Hole board test results: Thr92-Dio2 n = 7; Ala92-Dio2 n = 7; Thr92-Dio2 + T3 n = 7; Ala92-Dio2 + T3 n = 7. “% error” is the percentage of errors made by mice while recalling the holes that contain food. Values are expressed as mean ± SEM; Statistical analysis using 2-way ANOVA followed by Bonferroni’s test. ^a^ vs. Thr92-Dio2 with *p* < 0.0001; ^b^ vs. Ala92-Dio2 *p* < 0.0001; ^c^ vs. Thr92-Dio2 with *p* < 0.05; ^d^ vs. Thr92-Dio2 with *p* = 0.0028 and ^e^ vs. Ala92-Dio2 with *p* < 0.0007.

**Table 5:** Area and neuronal density of CA1, CA2, CA3 and Dentate gyrus (DG) hippocampal areas and retro splenial cortex (RSC) of 4-month-old female Ala92-Dio2 and Thr92-Dio2 mice.

Female					
	Area	Parameter	Thr92-Dio2	Ala92-Dio2	*p*-Value
	**CA1**	Area (mm^2^)	128.5 ± 7.78	100.1 ± 8.22	0.041
		Neuronal density (n/mm^2^)	7.5 ± 0.46	9.5 ± 0.05	0.008
	**CA2**	Area (mm^2^)	19.4 ± 1.93	16.53 ± 1.53	0.327
		Neuronal density (n/mm^2^)	6.9 ± 0.59	7.6 ± 0.29	0.480
**Hippocampus**					
	**CA3**	Area (mm^2^)	119.9 ± 10.1	95.17 ± 3.77	0.094
		Neuronal density (n/mm^2^)	7.7 ± 0.28	7.8 ± 0.08	0.657
	**DG**	Area (mm^2^)	175 ± 12.67	123.2 ± 19.21	0.046
		Neuronal density (n/mm^2^)	8.2 ± 1.04	9.7 ± 1.26	0.381
	**Ventral**	Neuronal density (n/mm^2^)	6,5 ± 0.35	6.4 ± 0.20	0.890
**RSC**					
	**Dorsal**	Neuronal density (n/mm^2^)	6.7 ± 0.36	6.2 ± 0.21	0.317

Values are expressed as mean ± SEM. Statistical analysis using Student’s *t*-test. (Thr92-Dio2 n = 6; Ala92-Dio2 n = 4); “n/mm^2^” is neurons/mm^2^; these experiments were repeated once with similar results; only one set of results in shown in the table.

## Data Availability

All statically significant results are shown in figures, tables, and supplementary tables, The raw data (RNA seq) will be provided upon request.

## Supplementary Materials

The following supporting information can be downloaded at: https://www.mdpi.com/article/10.3390/metabo12070629/s1, Table S1—Rna-seq genes from male Pfcx; Table S2—Rna-seq genes from male HC; Table S3—GSEA from male Pfcx; Table S4—GSEA from male HC; Table S5—GSEA Pfcx—select gene sets; Table S6—GSEA HC—select gene sets; Table S7—Rna-seq genes from female Pfcx; Table S8—Rna-seq genes from female HC; Table S9—GSEA from female Pfcx; Table S10—GSEA from female HC; Table S11—Overlaped genes in the heat map; Figure S1. Structural analysis of the left RSP cortex and left hippocampus of Ala92-Dio2 and Thr92-Dio2 of 4-mont-old female mice. (A) RSP from Ala92-Dio2 animals; (B) Hippocampus from Ala92-Dio2 animals; (C) RSP of Thr92-Dio2 animal; (D) Hippocampus from Thr92-Dio2 animal. RSPv indicates ventral retrosplenial cortex; RSPd indicates dorsal retrosplenial córtex. CA1 indicates Cornu Ammonis 1; CA2 indicates Cornu Ammonis; CA3 indicates Cornu Ammonis 3 and DG indicates Dentate gyrus. Bar scale: 200 µm.; Figure S2. Structural analysis of the left RSP cortex and left hippocampus of Ala92-Dio2 and Thr92-Dio2 of 7-mont-old male mice. (A) RSP from Ala92-Dio2 animals; (B) Hippocampus from Ala92-Dio2 animals; (C) RSP of Thr92-Dio2 animal; (D) Hippocampus from Thr92-Dio2 animal. RSPv indicates ventral retrosplenial cortex; RSPd indicates dorsal retrosplenial córtex. CA1 indicates Cornu Ammonis 1; CA2 indicates Cornu Ammonis; CA3 indicates Cornu Ammonis 3 and DG indicates Dentate gyrus; Bar scale: 200 µm.
