# Supplementary material for: Age Worsens the Cognitive Phenotype in Mice Carrying the Thr92Ala-DIO2 Polymorphism
Source: Metabolites. 2022 Jul 8;12(7):629. doi: 10.3390/metabo12070629 (PMC9319877; doi:10.3390/metabo12070629)
Supplement: Supplementary file 1 [file metabolites-12-00629-s001.zip › Figure S1 and Figure S2.pptx]

## Slide 1
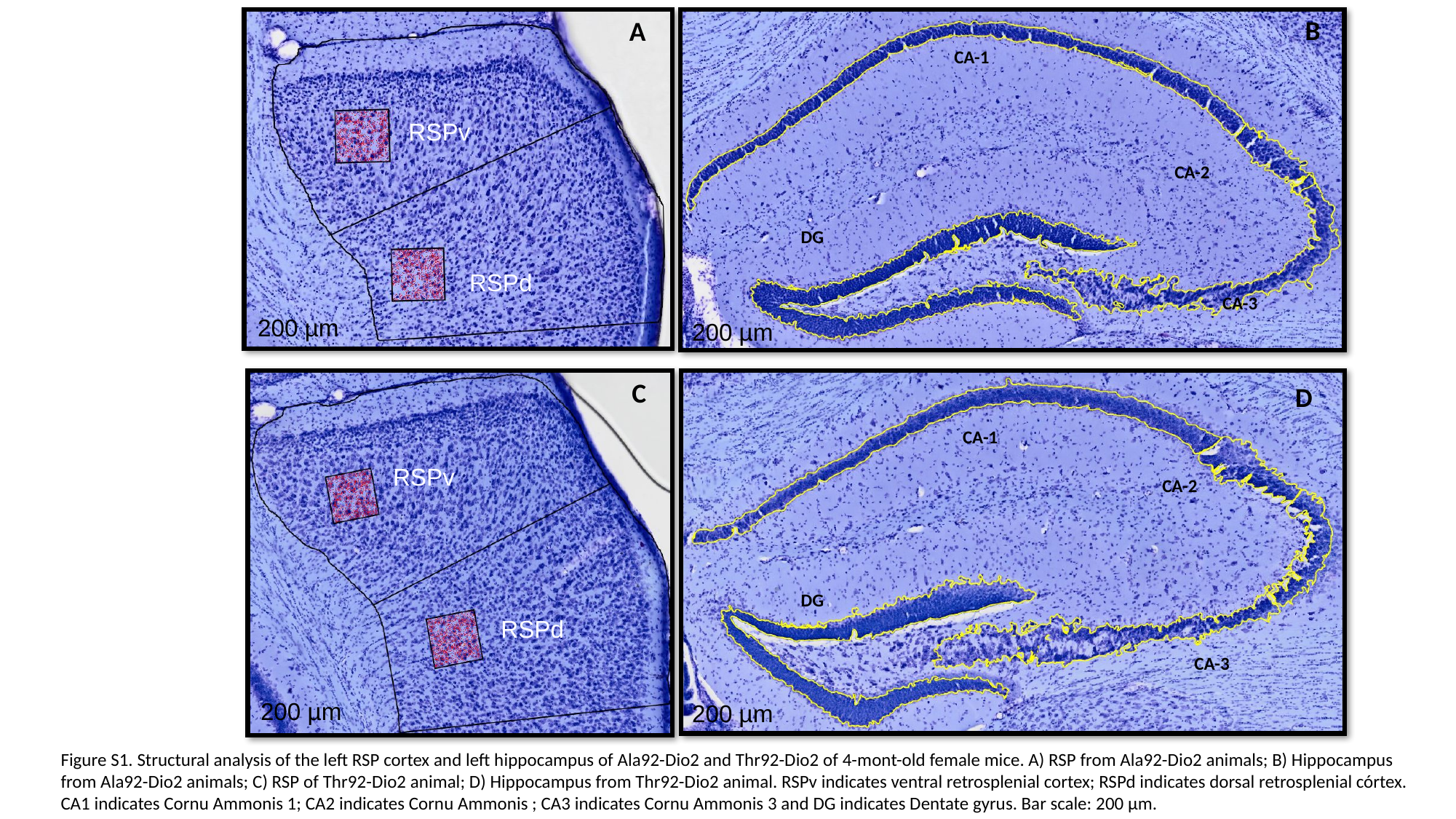

B
A
CA-1
RSPv
CA-2
DG
RSPd
CA-3
200 µm
200 µm
C
D
CA-1
RSPv
CA-2
DG
RSPd
CA-3
200 µm
200 µm
Figure S1. Structural analysis of the left RSP cortex and left hippocampus of Ala92-Dio2 and Thr92-Dio2 of 4-mont-old female mice. A) RSP from Ala92-Dio2 animals; B) Hippocampus from Ala92-Dio2 animals; C) RSP of Thr92-Dio2 animal; D) Hippocampus from Thr92-Dio2 animal. RSPv indicates ventral retrosplenial cortex; RSPd indicates dorsal retrosplenial córtex. CA1 indicates Cornu Ammonis 1; CA2 indicates Cornu Ammonis ; CA3 indicates Cornu Ammonis 3 and DG indicates Dentate gyrus. Bar scale: 200 µm.

## Slide 2
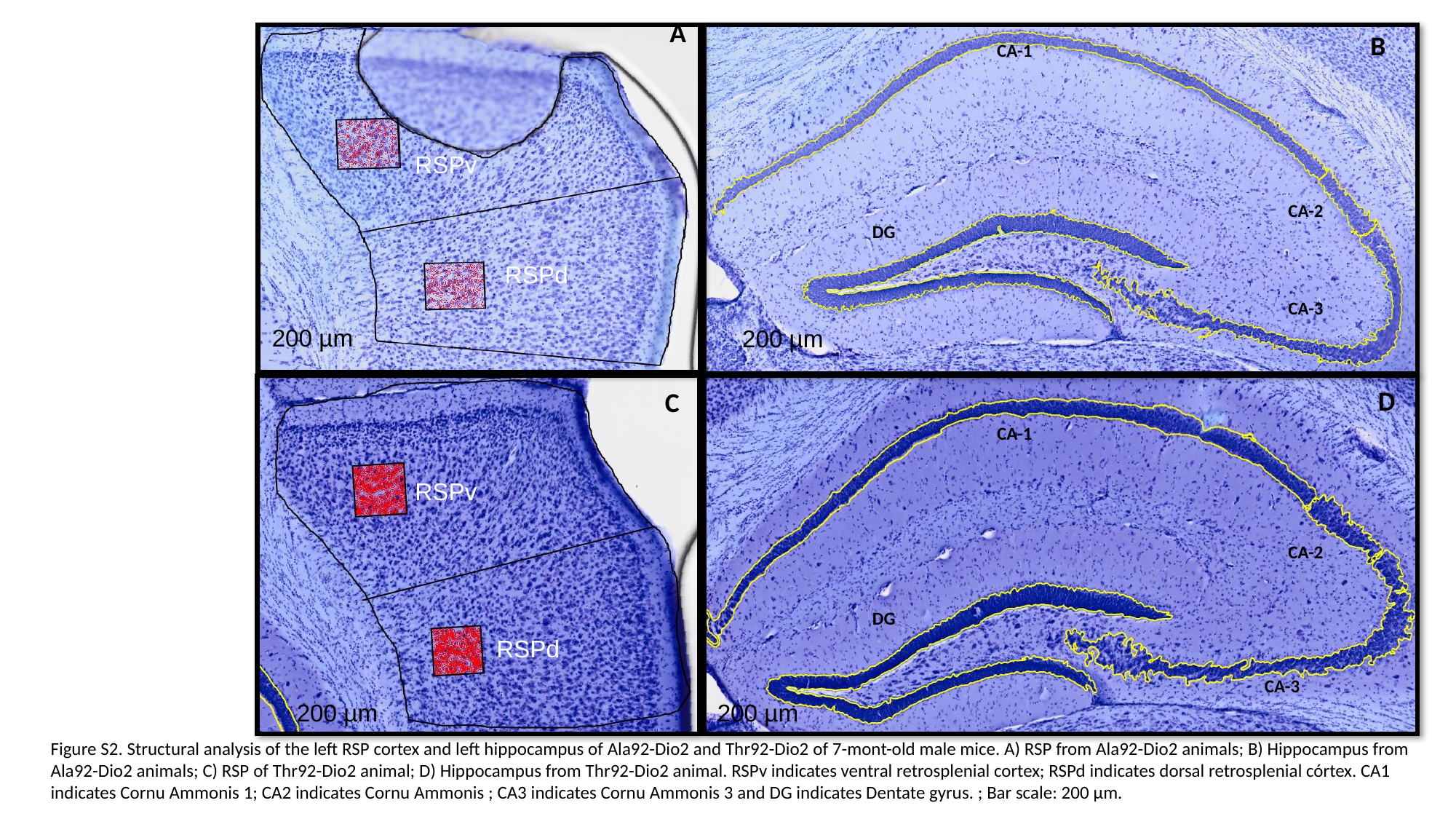

A
B
CA-1
RSPv
CA-2
DG
RSPd
CA-3
200 µm
200 µm
D
C
CA-1
RSPv
CA-2
DG
RSPd
CA-3
200 µm
200 µm
Figure S2. Structural analysis of the left RSP cortex and left hippocampus of Ala92-Dio2 and Thr92-Dio2 of 7-mont-old male mice. A) RSP from Ala92-Dio2 animals; B) Hippocampus from Ala92-Dio2 animals; C) RSP of Thr92-Dio2 animal; D) Hippocampus from Thr92-Dio2 animal. RSPv indicates ventral retrosplenial cortex; RSPd indicates dorsal retrosplenial córtex. CA1 indicates Cornu Ammonis 1; CA2 indicates Cornu Ammonis ; CA3 indicates Cornu Ammonis 3 and DG indicates Dentate gyrus. ; Bar scale: 200 µm.
