# Supplementary material for: Age Worsens the Cognitive Phenotype in Mice Carrying the Thr92Ala-DIO2 Polymorphism
Source: Metabolites. 2022 Jul 8;12(7):629. doi: 10.3390/metabo12070629 (PMC9319877; doi:10.3390/metabo12070629)
Supplement: Supplementary file 1 [file metabolites-12-00629-s001.zip › Table S11- Overlaped genes in the heat map.pdf]

Table S11- Overlapped genes differential expressed in AlaDio2 mice in the pre-frontal cortex and hippocampus of male and female mice

	Fold Change (Ala92Dio2 vs. Thr92Dio2)			
	**Pre-Frontal Cortex**		**Hippocampus**	
**Gene name**	**male**	**Female**	**Male**	**Female**
Gm17167	22.9	30.1	20.8	27.7
Gm49980	3.7	3.3	5.6	4.7
G530011O06Rik	3.1	1.9	3.7	2.5
Cd59a	2.5	2.5	1.9	1.9
Gm2163	2.3	3.1	1.5	2.1
D830030K20Rik	2.3	2.3	2.1	2.3
Gm37530	2.2	1.8	1.8	2.2
Fam205a4	2.2	2.2	2.3	2.6
Mid1	2.2	1.6	1.8	1.7
Gm21093	2.1	2.0	1.7	2.5
Gabra2	2.1	1.7	2.3	2.1
Wdfy1	2.0	1.5	2.2	1.6
Fam205a3	1.9	2.2	2.3	2.9
Gm43305	1.6	1.6	1.6	1.7
Gm20878	1.6	1.7	1.5	1.9
Ide	1.6	1.6	1.5	1.6
Btaf1	1.4	1.3	1.3	1.3
Zfp369	1.3	1.3	1.3	1.3
Cpne8	1.3	-1.2	1.0	1.3
Entpd4	1.3	1.4	1.3	1.3
Entpd4b	1.3	1.3	1.3	1.4
Tbc1d22a	1.1	1.2	1.1	1.1
Scg5	1.1	1.1	1.0	1.1
Gm11839	-1.3	-1.6	-1.2	-1.7
Bex1	-1.3	-1.4	-1.1	-1.3
Zfp975	-1.6	-1.5	-1.8	-1.5
Zfp976	-1.7	-1.5	-1.6	-1.6
Zfp951	-1.9	-2.2	-2.6	-2.1
Zfp97	-2.2	-1.7	-2.2	-1.9

Genes were selected using a Venn diagram from the input list of significant genes ($p<0.05$) in all categories (male PFC, female PFC; male HC and female HC); PFC: Pre-frontal cortex and HC: hippocampus.
