# Supplementary material for: Age Worsens the Cognitive Phenotype in Mice Carrying the Thr92Ala-DIO2 Polymorphism
Source: Metabolites. 2022 Jul 8;12(7):629. doi: 10.3390/metabo12070629 (PMC9319877; doi:10.3390/metabo12070629)
Supplement: Supplementary file 1 [file metabolites-12-00629-s001.zip › Table S5 - GSEA Pfcx - select gene sets.pdf]

Table S5- Gene sets enrichment analysis of differentially expressed genes in male and female Ala92-Dio2 pre-frontal cortex involved in brain metabolism and behavior.

Male				Female			
**Gene set**	**Description**	**Enrichment score**	**P-value**	**Gene set**	**Description**	**Enrichment score**	**P-value**
			**SIGNALING**				
GO:0045202	synapse	43	1.97E-19	GO:0061855	negative regulation of neuroblast migration	4	0.02
GO:0098978	glutamatergic synapse	29	3.53E-13	GO:0010977	negative regulation of neuron projection development	4	0.03
GO:0098916	anterograde trans-synaptic signaling	28	7.49E-13	GO:1904862	inhibitory synapse assembly	4	0.03
GO:0007268	chemical synaptic transmission	28	7.49E-13	GO:0050808	synapse organization	3	0.03
GO:0099536	synaptic signaling	27	1.42E-12	GO:0050807	regulation of synapse organization	3	0.04
GO:0099537	trans-synaptic signaling	27	1.42E-12	GO:0099558	maintenance of synapse structure	3	0.04
GO:0014069	postsynaptic density	14	6.13E-07	GO:0051965	positive regulation of synapse assembly	3	0.05
GO:0050806	positive regulation of synaptic transmission	13	2.31E-06	GO:0099181	structural constituent of presynapse	3	0.05
GO:0032228	regulation of synaptic transmission, GABAergic	11	1.13E-05	GO:0098981	cholinergic synapse	3	0.05
GO:0001508	action potential	10	5.35E-05	GO:1903543	positive regulation of exosomal secretion	3	0.05
GO:0060291	long-term synaptic potentiation	9	1.59E-04	GO:0005165	neurotrophin receptor binding	3	0.05
GO:0050808	synapse organization	8	2.92E-04	GO:0048011	neurotrophin TRK receptor signaling pathway	3	0.05
GO:0019228	neuronal action potential	8	3.08E-04	GO:0005165	neurotrophin receptor binding	3	0.05
GO:0060079	excitatory postsynaptic potential	8	3.84E-04				
GO:0098984	neuron to neuron synapse	8	4.46E-04				
GO:0098982	GABA-ergic synapse	7	7.10E-04				
GO:1900273	positive regulation of long-term synaptic potentiation	7	8.93E-04				
GO:0098900	regulation of action potential	6	2.10E-03				
GO:0004935	adrenergic receptor activity	5	0.01				
GO:2000310	regulation of NMDA receptor activity	4	0.02				
GO:2000311	regulation of AMPA receptor activity	4	0.02				

GO:0051932	synaptic transmission, GABAergic	4	0.03				
GO:1990416	cellular response to brain-derived neurotrophic factor stimulus	3	0.04				
GO:0051967	negative regulation of synaptic transmission, glutamatergic	3	0.04				
GO:0071875	adrenergic receptor signaling pathway	3	0.04				
				**NEUROPLASTICITY**			
GO:0030425	dendrite	23	1.51E-10	GO:1990769	proximal neuron projection	5	0.01
GO:0044306	neuron projection terminus	17	2.67E-08	GO:0045665	negative regulation of neuron differentiation	4	0.02
GO:0050877	nervous system process	13	1.86E-06	GO:0045665	negative regulation of neuron differentiation	4	0.02
GO:0044309	neuron spine	12	4.56E-06	GO:0045665	negative regulation of neuron differentiation	4	0.02
GO:0043197	dendritic spine	12	6.88E-06	GO:1904800	negative regulation of neuron remodeling	4	0.02
GO:0045664	regulation of neuron differentiation	9	1.43E-04	GO:1904799	regulation of neuron remodeling	4	0.02
GO:0050767	regulation of neurogenesis	8	2.44E-04	GO:0061853	regulation of neuroblast migration	4	0.02
GO:0010976	positive regulation of neuron projection development	8	3.63E-04	GO:0061855	negative regulation of neuroblast migration	4	0.02
GO:1900006	positive regulation of dendrite development	8	3.74E-04	GO:0010977	negative regulation of neuron projection development	4	0.03
GO:0031644	regulation of nervous system process	8	4.43E-04	GO:0003357	noradrenergic neuron differentiation	3	0.05
GO:0050769	positive regulation of neurogenesis	8	4.73E-04				
GO:0097106	postsynaptic density organization	8	5.42E-04				
GO:0045666	positive regulation of neuron differentiation	7	8.64E-04				
GO:0048812	neuron projection morphogenesis	7	9.11E-04				
GO:0021891	olfactory bulb interneuron development	6	2.28E-03				

GO:0050771	negative regulation of axonogenesis	6	2.98E-03
GO:0097485	neuron projection guidance	6	3.25E-03
GO:0060999	positive regulation of dendritic spine development	5	0.01
GO:0048169	regulation of long-term neuronal synaptic plasticity	5	0.01
GO:0007409	axonogenesis	5	0.01
GO:1990138	neuron projection extension	5	0.01
GO:0021542	dentate gyrus development	4	0.02
GO:0097449	astrocyte projection	4	0.02
GO:0007270	neuron-neuron synaptic transmission	4	0.02
GO:1905809	negative regulation of synapse organization	4	0.02
GO:0050775	positive regulation of dendrite morphogenesis	4	0.02
GO:0019227	neuronal action potential propagation	4	0.03
GO:0097484	dendrite extension	4	0.03
GO:0050774	negative regulation of dendrite morphogenesis	4	0.03
GO:0099563	modification of synaptic structure	3	0.04

COGNITION

GO:0007610	behavior	19	5.23E-09
GO:0007611	learning or memory	11	1.92E-05
GO:0007612	learning	10	3.26E-05
GO:0050890	cognition	10	3.78E-05
GO:0050890	cognition	10	3.78E-05
GO:0007626	locomotory behavior	10	6.08E-05
GO:0008306	associative learning	9	1.10E-04
GO:0050795	regulation of behavior	8	3.76E-04
GO:0008542	visual learning	7	6.77E-04

APOPTOSIS

GO:2001272	positive regulation of cysteine-type endopeptidase activity involved in execution phase of apoptosis	4	0.02
GO:1900117	regulation of execution phase of apoptosis	3	0.03

GO:0007632	visual behavior	7	8.97E-04
GO:0007613	memory	5	0.01
GO:0035640	exploration behavior	5	0.01
GO:0002209	behavioral defense response	4	0.02
GO:0042755	eating behavior	4	0.02
GO:0007616	long-term memory	3	0.03
GO:0007616	long-term memory	3	0.03
GO:0040012	regulation of locomotion	3	0.05

Analysis done using the Partek Flow package. Gene set column contains Gene Ontology identifiers. Enrichment score: negative natural logarithm of the enrichment P-value derived from the Fisher's exact test. The higher the enrichment score, the more overrepresented the GO category is within the input list of significant genes; only gene sets with a $p<0.05$ are shown.
