# Supplementary material for: Age Worsens the Cognitive Phenotype in Mice Carrying the Thr92Ala-DIO2 Polymorphism
Source: Metabolites. 2022 Jul 8;12(7):629. doi: 10.3390/metabo12070629 (PMC9319877; doi:10.3390/metabo12070629)
Supplement: Supplementary file 1 [file metabolites-12-00629-s001.zip › Table S6 - GSEA HC - select gene sets.pdf]

Table S6- Gene sets enrichment analysis of differentially expressed genes in male and female Ala92-Dio2 hippocampus involved in brain metabolism and behavior.

Male				Female			
**Gene set**	**Description**	**Enrichment score**	**P-value**	**Gene set**	**Description**	**Enrichment score**	**P-value**
				**NEUROPLASTICITY**			
GO:0038179	neurotrophin signaling pathway	4	0.02	GO:0097475	motor neuron migration	6	2.56E-03
GO:0008038	neuron recognition	4	0.02	GO:0021859	pyramidal neuron differentiation	4	0.02
GO:0021956	central nervous system interneuron axonogenesis	4	0.02	GO:0048011	neurotrophin TRK receptor signaling pathway	4	0.02
GO:0031548	regulation of brain-derived neurotrophic factor receptor signaling pathway	3	0.04	GO:0097476	spinal cord motor neuron migration	3	0.05
				**BEHAVIOR**			
GO:1902437	positive regulation of male mating behavior	3	0.04	GO:0002125	maternal aggressive behavior	3	0.05
GO:0007625	grooming behavior	3	0.04				
				**APOPTOSIS**			
GO:1901216	positive regulation of neuron death	3	0.05	GO:0097199	cysteine-type endopeptidase activity involved in apoptotic signaling pathway	9	1.42E-04
GO:0070997	neuron death	3	0.03	GO:0097153	cysteine-type endopeptidase activity involved in apoptotic process	7	6.39E-04
GO:0043525	positive regulation of neuron apoptotic process	4	0.03	GO:0031264	death-inducing signaling complex	6	3.79E-03
				GO:0097194	execution phase of apoptosis	4	0.01
				GO:0090389	phagosome-lysosome fusion involved in apoptotic cell clearance	4	0.02
				GO:0006919	activation of cysteine-type endopeptidase activity involved in apoptotic process	4	0.03
				GO:0043280	positive regulation of cysteine-type endopeptidase activity involved in apoptotic process	3	0.04
				**INFLAMMATION**			
GO:1900017	positive regulation of cytokine production involved in inflammatory response	3	0.05	GO:0072559	NLRP3 inflammasome complex	5	0.01

GO:0044546	NLRP3 inflammasome complex assembly	3	0.04	GO:0061702	inflammasome complex	4	0.03
GO:0035490	regulation of leukotriene production involved in inflammatory response	4	0.02				
GO:0040012	regulation of locomotion	3	0.05				
	**SIGNALING**						
GO:0031841	neuropeptide Y receptor binding	4	0.02				
GO:0006836	neurotransmitter transport	4	0.03				
GO:0005165	neurotrophin receptor binding	3	0.04				
GO:0021894	cerebral cortex GABAergic interneuron development	3	0.04				

Analysis done using the Partek Flow package. Gene set column contains Gene Ontology identifiers. Enrichment score: negative natural logarithm of the enrichment P-value derived from the Fisher's exact test. The higher the enrichment score, the more overrepresented the GO category is within the input list of significant genes; only gene sets with a p<0.05 are shown.
